# Impact of a Pandemic and Remote Learning on Team Development and Elements of Compassion in Prospective Medical Students Taking a Medical Humanities Course

**DOI:** 10.3390/ijerph18094856

**Published:** 2021-05-02

**Authors:** Lon J. Van Winkle, Brian D. Schwartz, Alexis Horst, Jensen A. Fisher, Nicole Michels, Bradley O. Thornock

**Affiliations:** 1 Department of Medical Humanities, Rocky Vista University, 8401 S. Chambers Road, Parker, CO 80134, USA; bschwartz@rvu.edu (B.D.S.); ahorst@rvu.edu (A.H.); jfisher@rvu.edu (J.A.F.); nmichels@rvu.edu (N.M.); 2 Department of Medical Humanities, Rocky Vista University, 255 E. Center Street, Ivins, UT 84738, USA; bthornock@rvu.edu

**Keywords:** compassion, COVID-19 pandemic, implicit bias, reflective capacity, remote learning, team-based learning

## Abstract

Introduction: We reported previously that when teams of students reflect on readings about communication, unconscious bias, and service-learning, their critical reflection, implicit bias mitigation, empathy, and compassionate behavior all increase. However, would these gains occur when intimate classroom settings, in-person team meetings, and direct interactions with people served were lost owing to the COVID-19 pandemic and remote learning? Methods: Before an online Medical Humanities course began in August 2020 and following the course in December 2020, 61 prospective medical students (54.1% female) completed reliable surveys of their reflective capacity (RC) and cognitive empathy (compassion). Students also completed surveys about their implicit biases and team community service in December 2020. Results: Both RC and empathy scores increased in students after they reflected on difficulties in communication, unconscious biases, and team service-learning experiences in the course. In written reflections, they reported how their compassionate behavior also grew owing to bias mitigation. Most students concurred that “unconscious bias might affect some of (their) clinical decisions or behaviors as a healthcare professional” and vowed to remain aware of these biases in clinical settings. Conclusions: Compared to previous years, the pandemic and remote learning had minimal effects on the benefits of our Medical Humanities course.

## 1. Introduction

Healthcare professional trainees should learn to grow their compassion, because more compassion likely increases patient well-being and reduces provider burn-out [1,2,3]. It was suggested that compassion can be “animated” by humanitarian principles [4], including accountability, excellence, responsibility, altruism, integrity, and regard for other people [5]. Such values can be applied when students reflect on real experiences [6,7,8], but how are the values put in motion when experiences are dampened by a pandemic and largely remote learning? For example, we cultivated more compassionate behavior in teams of pharmacy and medical students who performed service-learning [9,10], but do these results change when personal contact is largely precluded?

In more recent studies of prospective medical student teams in a Medical Humanities course, we also asked the teams to select and perform community service. These assignments led students to experience mental difficulties and to resolve the difficulties, including resolving their implicit biases through critical reflection [7,8]. In such reflection, one “recognizes how their thoughts and behaviors do not match their personal and humanistic values, experiences perplexity, doubt, hesitation, or mental difficulties (i.e., dissonance), and decides how better to align their values, thoughts, and behaviors (i.e., dissonance reconciliation)” [10,11,12]. Consequently, students reported more compassionate behavior toward people they were serving in their written reflections on service-learning.

Our students also developed highly favorable opinions about team-based learning, their teams, service-learning, and unconscious bias mitigation [7,8]. Moreover, this emphasis on implicit bias led students to see that unconscious bias might influence their decisions as future clinicians, and they wanted to guard against this influence of bias. Likewise, reflections on team community service were accompanied by higher reflective capacity (RC) and cognitive empathy (compassion) scores by students [7,8], as measured by reliable and validated surveys of these characteristics [13,14,15]. Finally, asking students to reflect on how community service relates to their basic science courses apparently led most of them to show more interest in studying for the courses [7,8].

All these results were, however, obtained in association with intimate classroom settings, in-person team meetings outside of class, and direct interactions with people served in our community. As a result of the COVID-19 pandemic, all these learning activities were conducted remotely in our Medical Humanities course for the 2020 cohort of prospective medical students. According to Keating [16], when we see people in person, rather than virtually, and observe them watching us, it helps us “establish trust, gain commitment, confirm understanding and consensus, and understand emotional states.” While interactive online courses appear to be more effective than less interactive approaches [17,18], we anticipated that interaction in person would still be most successful. Moreover, Williamson and associates [19] cautioned that educational technologies may not be a ready-made remedy for learning during the COVID-19 pandemic. For these reasons, we predicted that some or all the benefits of our course would be lost or dampened owing to remote learning. Our specific hypotheses were as follows.

In contrast to results with three prior cohorts of prospective medical students [7,8], the COVID-19 pandemic and remote learning will diminish the ability of a Medical Humanities course to foster the following in a new cohort of students.

1. In prior studies we observed development of highly positive attitudes toward service-learning, bias mitigation, and the value of teammates in helping to foster more compassionate behavior. In comparison, we predicted that difficulties in forming relationships and performing team service-learning remotely would temper this growth in a new cohort of students.
2. When class discussions, team meetings, and service were in person, we found RC scores to increase in association with students’ reflection for our Medical Humanities course. Contrary to this result, we expected students’ RC scores not to increase, owing to the remote-learning environment. In particular, we thought the increase in the “reflection with others” component of RC [8] would be lost from the online course.
3. Cognitive empathy scores also increased during our prior, in-person course, but we expected scores would not increase in students during remote learning, owing, in part, to their correlation with RC scores.
4. When learning in person, students came to realize that their future decisions as clinicians might be influenced by unconscious bias, but we felt remote learning would interfere with this realization.
5. When working together on campus, students came to appreciate that service-learning experiences increased their interest in studying for all their courses. Remote-learning and the COVID-19 epidemic was expected to distract students from gaining this appreciation.

## 2. Methods

### 2.1. Participants

Sixty-one prospective medical students participated in this study in 2020, and they were compared to three previous cohorts of students in our Medical Humanities course (a total of 83 students from 2017 to 2019). Each cohort was studied from August through December of the year they were enrolled in the Rocky Vista University (RVU) Master of Science in Biomedical Sciences (MSBS) program. In 2020, 41 students matriculated at the RVU campus in Parker, Colorado, US, and 20 students attended RVU at the Ivins, Utah, US campus. Students’ ages ranged from 21 to 42 years (mean of 25.8 years), 45.9% identified as male, 54.1% were white, 19.7% described themselves as Asian, 11.5% identified as Hispanic, 9.8% were Black/African American, and 4.9% were undisclosed. Owing to the COVID-19 pandemic, learning in the course was remote (i.e., not in person), and students were not required to move to Parker or Ivins. While Zoom and similar video conferencing platforms provide many tools [20], we used only the screenshare and breakout rooms features. Teams of students were asked to remain in gallery mode in breakout rooms while taking team quizzes and holding team discussions in class, and screenshare was sometimes used by instructors in class session wrap-up discussions during the final 10 to 15 min of the 50 min class sessions.

The humanities course was intended to foster bias mitigation and develop communication skills in students using quizzes on readings, implicit association testing, classroom application exercises, and written reflections on service-learning projects in a team-based learning (TBL) format [21,22]. Our readings on communication and bias in 2020 were from four pertinent books used when the humanities course was in-person [7,8] and, thus, could not involve interactive e-textbooks [23] if comparison of remote to in-person learning was to remain as legitimate as possible. Similarly, while interactive testing via computers appears to be at least as effective as traditional written evaluation [24], the provision of feedback using scratch off answer cards fosters information retention better than the same feedback via computers [25]. Hence, we mailed the TBL scratch off cards [21,22] to one member of each team prior to team quizzes. A few students were able to perform some service-learning in person during the first half of the course, although students could not do so as teams, and all students were prohibited from in-person service as of October 2020 owing to the worsening pandemic.

### 2.2. Team Formation

Teams of four to six students were formed utilizing several criteria identified by students prior to the start of the MSBS program in August 2020. In the virtual Zoom classroom, students worked in their teams to complete team quizzes and application exercises concerning communication skills and implicit bias. Outside of class, teams used Zoom and other virtual methods of their choice to select and perform service-learning projects [7,8].

A minimum of five hours of service were required of each team member, and each team member worked alone and largely remotely. Teams first met virtually for about 90 min to select and plan their project and then at least three more times to discuss each team member’s experiences with the project and its overall progress. At meeting times convenient for all members, teams discussed individual students’ written critical reflections about planning and performing service-learning.

Teams used meetings to help write reflections and minutes of their meetings. In their written reflections, they also connected their service-learning projects to other MSBS courses. For example, many teams considered the possible physiological consequences of being homeless as they studied for their Physiology course. Only this expectation to connect service-learning to other courses, and the description of critical reflection below, were given to students to allow them maximum flexibility in deciding how best to perform self-examination and introspection in their teams.

At least one page of reflections was written by each student for every two hours of service to the community, and team written observations were generated from every team meeting. Students earned one credit-hour for completing the humanities course, and 52% of their grades were determined using the four sets of individual and team written reflections and team meeting minutes. The course director received these writing assignments at four- to five-week intervals during the 17-week semester.

### 2.3. Assessment of Written Reflections for Dissonance and Critical Reflection

Writing assignments were appraised for critical reflection on dissonance, its reconciliation, and bias against various groups of people [7,8,12,26,27]. Self-examination (including resultant compassionate behavior) was defined as “when a student recognized how their thoughts (and actions) did not match their personal and humanistic values; experienced perplexity, doubt, hesitation, or mental difficulties (i.e., dissonance); and began to decide how better to align their thoughts (and behaviors) with their values (i.e., dissonance reconciliation). That is, dissonance reconciliation had to be present for self-examination (and compassionate behavior) to be present” [7,8,10,11,12]. Nguyen and associates’ model of reflective activity is consistent with this definition [28].

Typically, grades ranged from 100% (self-examination shown) to 80% (writing without self-examination). A score of 90% was earned for dissonance without its reconciliation. As described previously, different faculty members give similar grades for these written critical reflections (*r* = 0.92 [27]).

### 2.4. Quantitative Surveys to Measure RC, Cognitive Empathy, and Opinions about Implicit Bias and Team- and Service-Learning

Students were invited to complete an online version of the Reflective Practice Questionnaire (RPQ) [13,14] during the first session of the Medical Humanities course in August 2020 and at the final session in December 2020, to determine whether increases in RC scores were associated with reflections performed for the course. The RPQ is intended for use with various groups of people from healthcare professionals to the general public [13,14]. It is a self-report instrument composed of 40 items, 16 of which measure reflective capacity (RC). Subcomponents of RC include reflection-in-action, reflection-on-action, reflection with others, and self-appraisal [13,14]. Other theoretically pertinent constructs measured by the RPQ include desire for improvement, general confidence, confidence communicating, uncertainty or stress interacting with patients/clients, and job satisfaction. Each of the latter sub-scales are assessed using four items. The current version of the RPQ has a response scale from 1 (not at all) to 6 (extremely) with intermediate responses of 2 (slightly), 3 (somewhat), 4 (moderately), and 5 (very much). The RPQ is quite reliable. Its Cronbach’s alpha values range from 0.75 to 0.91 for the RPQ subscales [13,14]. The RPQ can be found online in Rogers and associates [14].

At the same times as completion of the RPQ, and with permission of the copyright holder (© Thomas Jefferson University, 2001, all rights reserved), students also completed an online version of the Jefferson Scale of Empathy (JSE, HPS-Version; Cronbach’s alpha reliability values of about 0.84 [15,29]; current version online in Fjortoft et al. [29]). The 20-item JSE has been shown in numerous studies to be a valid and reliable measure of cognitive empathy in healthcare professional students and practitioners (reviewed in [15]). Up to three factors (perspective taking, compassionate care, and, sometimes, standing in the patient’s shoes) emerge in studies with the JSE.

Finally, students responded to an online survey about their opinions concerning implicit bias, team-based learning, and service to the community in December 2020 (Table 1). Students were also given the option to comment on each item in this survey, and their written responses could be categorized into two or three sets of similar answers as shown beneath each item in Table 1. Surveys were not administered by the authors but by an administrative assistant in association with class sessions dedicated to survey completion. After pairing RPQ and JSE pre- and post-survey responses using randomly assigned numbers to each student, all records of students’ names and their completed surveys were destroyed by the administrative assistant to maintain students’ anonymity. Data were tabulated and analyzed by one of us (LJV) beginning in December 2020. Response rates for each survey were 100%, although some students did not respond to every item, so the number of responses in Table 1 is usually less than 61.

### 2.5. Statistical Analysis

GraphPad Prism 8.3.0 Software, Inc. (La Jolla, CA, USA) was used to perform statistical analyses, and *p* values less than 0.05 were considered statistically significant. Whether students’ attitudes were neutral toward implicit bias, team-based learning, and community service was determined with nonparametric, one-sample Wilcoxon tests, since the distributions of responses in Table 1 are not normally distributed. Moreover, we determined whether the median frequencies of students’ responses in 2020 (Table 1) differed from the responses of students in 2017–2019 using unpaired Mann–Whitney tests. In the case where each of the four years were compared to one another (item 8 in Table 1), the Kruskal–Wallis test with multiple comparisons was used to determine whether they differed significantly. In the latter case, we also used the Mann–Whitney test to compare numerically adjacent medians. Statistically significant outlier values in each set of data were detected using ROUT (Q = 1%).

Whether students’ JSE and RC scores increased during the Medical Humanities course was determined with paired *t*-tests. In general, these data were normally distributed, although paired *t*-tests do not require such distributions. Effect sizes as *r* values were calculated during *t*-tests using the GraphPad Prism 8.3.0 Software [30]. With the same software, Pearson *r* values were calculated to detect correlations between each set of data in the surveys.

The Rocky Vista University Institutional Review Board (IRB) assessed this study and categorized it as exempt according to pertinent criteria. Students gave written informed consent to publish their written critical reflections.

## 3. Results

### 3.1. Students’ Attitudes after Remote Versus in-Person Learning

In 2020, students appeared to be somewhat less enthusiastic than in prior years about service-learning and whether it helped them to mitigate their biases (items 1, 3, 9, and 10 in Table 1). For example, while the median responses to item 3 in Table 1 were 7.0 (strongly agree) in both 2020 and 2017–2019, the proportion of lower responses were highly skewed toward 6.0 (agree) in 2017–2019. Such was not the case, however, in 2020 (item 3 in Table 1). According to the nonparametric Mann–Whitney test, this difference was statistically significant (*p* < 0.05). Nevertheless, most students in 2020 felt community service experiences were beneficial to them and helped them to see their potential biases against others (Table 1 and Table 2). Students also expressed favorable opinions about team-based learning in 2020 (item 6 in Table 1), and affection for their teams was even more pronounced than in prior years (item 2 in Table 1).

Remote discussion of implicit association test results and other evidence of bias also appeared to be somewhat less impactful in helping students realize that “unconscious bias might affect some of (their) clinical decisions or behaviors as healthcare professionals” (item 11 in Table 1). Nevertheless, 82% of students agreed with this statement (item 11) in 2020. Similarly, the remote-learning environment and COVID-19 epidemic appeared to keep some students from appreciating how service-learning experiences can cause them “to study for all (their) courses with more interest” (item 8 in Table 1).

### 3.2. Improvements in Students’ RC and Empathy Scores in 2020

**Figure 1:**
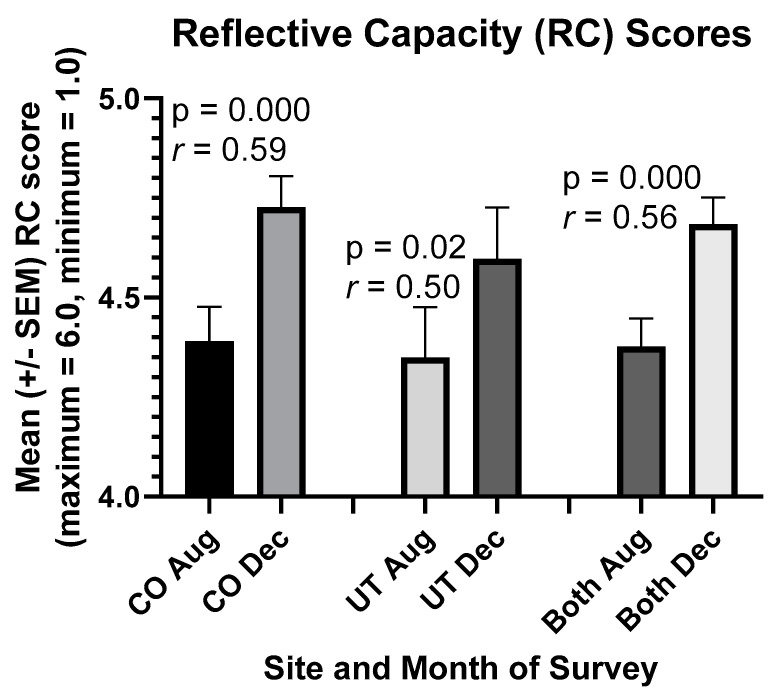
Students’ reflective capacity (RC) scores rose during their Medical Humanities course in 2020. Paired *t*-tests were used to compare means in Aug and Dec. *n* = 41 students in Colorado, 20 students in Utah, and 61 students combined (i.e., both).

**Figure 2:**
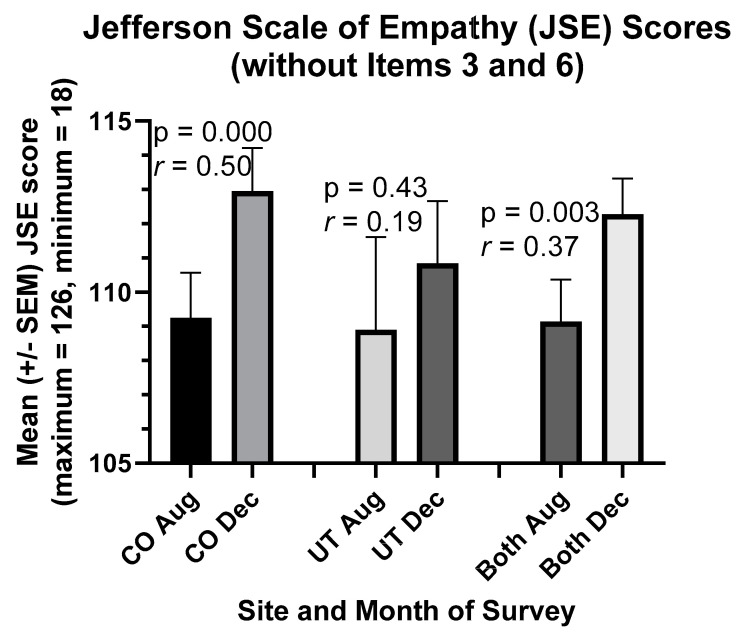
Students’ cognitive empathy (JSE) scores rose during their Medical Humanities course in 2020. Paired *t*-tests were used to compare means in Aug and Dec. *n* = 41 students in Colorado, 19 students in Utah, and 60 students combined (i.e., both). One student’s values were statistically significant outliers in Utah.

RC scores rose significantly in students in association with reflection on team service-learning experiences in our online course (Figure 1). Such was the case for Colorado and Utah students separately and combined (*p* = 0.000). In particular, the “reflection with others” component of RC increased significantly (*r* = 0.32, *p* = 0.01) despite remote learning. Similarly, students’ cognitive empathy scores increased in the remote-learning environment between August and December 2020 (Figure 2, *p* = 0.003). RC and cognitive empathy scores were highly correlated in both Colorado and Utah students in August (Colorado, *r* = 0.71; Utah, *r* = 0.69) and December (Colorado, *r* = 0.56; Utah, *r* = 0.73).

## 4. Discussion

### 4.1. Remote Learning Was Nearly as Effective in Promoting Student Growth as Learning in-Person

Our data partially support a few of our hypotheses. First, difficulties in forming relationships and performing team service-learning remotely did seem to temper development of positive attitudes toward community service (items 1 and 3 in Table 1) and recognizing/mitigating implicit biases (items 9 and 10 in Table 1), although students still made considerable progress in these ways (Table 1 and Table 2). Students also expressed favorable opinions about team-based learning (item 6 in Table 1), and affection for their teams was even more pronounced than in prior years (item 2 in Table 1).

Regarding our hypothesis “4” in the Introduction, remote discussion of implicit association test results and other evidence of bias appeared to be somewhat less impactful in helping students realize that “unconscious bias might affect some of (their) clinical decisions or behaviors as healthcare professionals” (item 11 in Table 1). Nonetheless, 82% of students agreed with this statement (item 11) in 2020. Likewise, the remote-learning environment and COVID-19 epidemic appeared to distract some students from appreciating how service-learning experiences can cause them “to study for all (their) courses with more interest” (item 8 in Table 1).

In contrast, however, RC scores rose significantly in students owing to reflection on team service-learning experiences (Figure 1), as was the case in our prior studies [7,8]. Such was the case for both Colorado and Utah students in 2020, with an overall effect size of crucial practical importance (*r* = 0.56). Notably, the “reflection with others” component of RC increased significantly (*r* = 0.32, *p* = 0.01) despite the difficulties in forming relationships we predicted.

Likewise, students’ cognitive empathy scores (a component of compassion) increased by a moderate to crucial amount in the remote-learning environment between August and December 2020 (*r* = 0.37, Figure 2). While the increase in empathy scores among Utah students was not statistically significant, it resembled the increase in RC scores in Utah (Figure 1 and Figure 2). Interestingly, as in our prior study with Colorado students [8], “desire for Improvement,” as measured by the RPQ, became highly correlated with both RC scores (*r* = 0.62) and cognitive empathy scores (*r* = 0.50) in Colorado students in December, but no such change or correlation was observed in Utah students. Nevertheless, RC and cognitive empathy scores were highly correlated in both Colorado and Utah students in August (Colorado, *r* = 0.71; Utah, *r* = 0.69) and December (Colorado, *r* = 0.56; Utah, *r* = 0.73), as was the case in our prior studies with Colorado students [7,8].

As explained in our previous paper [8], we did not include items 3 and 6 of the JSE in our analysis of the empathy scores in Figure 2 because we emphasize in our course how difficult it is (albeit particularly important) to “stand in the patient’s shoes.” Items 3 and 6 of the JSE comprise this third (“standing in the patient’s shoes”) of three factors sometimes measured by the JSE [31,32]. Nevertheless, cognitive empathy scores increased significantly in students between August and December, even when these items were included in the analysis for the complete set of students (*r* = 0.25, *p* < 0.05). Moreover, the full cognitive empathy scores remained highly correlated with RC scores in both August (*r* = 0.71) and December (*r* = 0.61).

A separate contributing factor to our results may have been the growing concern regarding diversity, equity, and inclusion (DEI) following the death of George Floyd in May of 2020. While formal presentations concerning DEI did not begin at our university until January 2021 and after completion of our course, the Black Lives Matter movement and similar social concerns were sometimes mentioned by teams of students in written critical reflections. Indeed “#WhiteCoatsforBlackLives and #ShutdownSTEM are highly visible exhortations to raise awareness of racism on the campuses of academic medical centers” [33]. For all the reasons above, remote learning during a pandemic had minimal effects on the important growth toward greater compassion fostered during our Medical Humanities course.

### 4.2. Can Our Approach Be Implemented More Widely?

Implicit bias remains a troublesome worldwide problem [34,35,36,37,38]. Healthcare professionals hold negative attitudes toward various groups of people (Table 2) and discriminate against them. Such biases lead to less-than-optimal patient care. Similarly, patients may be denied the best possible healthcare owing to implicit biases toward applicants in residency selection [39,40]. However, implicit bias mitigation could be fostered in all prospective healthcare professionals using strategies like those described here. Even in a pandemic and using remote learning in 2020, only one of 61 students stated that unconscious bias will not affect their behaviors as healthcare professionals, while 30 students commented that implicit bias will affect their behaviors, but they will work to control the bias and behaviors (item 11 in Table 1). If employed throughout practitioners’ careers, such learning could improve care for marginalized people and foster public health. Both within and outside of their practices, healthcare providers should use critical reflection to mitigate unconscious biases throughout their careers.

Implicit association tests assisted us in helping students mitigate biases against people based on their body weight, gender, sexual orientation, and race. Through experiences in their service-learning projects, students expanded this list to encompass younger, older, homeless, and economically disadvantaged people (Table 2). Hence, a need to mitigate bias against groups of people expands depending on the situation in which people are served. To emphasize this important perspective, our students watched the final 60 min of a Gates Foundation video showing ways to mitigate unconscious biases and improve healthcare in various countries of the world and accessed for class on 6 November 2020. (The Gates Foundation video can be found at https://youtu.be/1SbUSj5iEgs, accessed on 6 November 2020).

Our approach is similar to a “pedagogy of discomfort” [41,42], and our data reflect Sukhera and associates’ “transformative learning model” [43]. They found that a disorienting experience, such as a pandemic, unanticipated remote learning, service-learning, or new awareness of implicit bias, causes critical reflection, as we observed in our students. Then, our students labored to improve their communication skills and show more compassion when serving others (as is also illustrated in Figure 1 of [43]). The latter authors propose their transformative learning model to fight negative unconscious biases toward patients and other healthcare professionals [43].

To establish such training as convention, we should include these efforts in other courses and rotations for healthcare professional students. At our university, we convinced some faculty members, administrators, and, importantly, most students in our course that critical reflection on community service fostered more compassionate behavior. These members of the faculty then began to include some team activities in their courses. In these other courses, such as Pharmacology and Physiology, the same teams that were formed for work in Medical Humanities performed course projects together and related their service-learning experiences to activities in those other courses.

Eventually, all faculty members should adopt plans to incorporate written critical reflections on service-learning into their courses and to assess these aspects of student development. The Interprofessional Education course for physician assistant and medical students and the Immunology course for MSBS students at our institution have already adopted this plan. Emerging data show that service-learning and related experience can lead students to study with more interest for all the courses in which they are enrolled, at least when learning is in person (item 8 in Table 1 for 2019). Both non-cognitive and academic growth are fostered in students through written critical reflections on service-learning experiences [44,45,46,47]. A mechanism exists at our university to establish as convention these methods to help train all healthcare students in basic science courses, since most faculty members teach similar courses in different programs.

To continue the program in subsequent clinical science rotations, we could enlist the help of students. Students influence the curriculum and environment at our institution, particularly in relation to their clinical rotations. Students would expect a vigorous program that fosters growth of their compassion during the first one or two years of their training to continue into their clinical years. They would also want the strong support from their teammates to continue during challenging new clinical rotations. (See items 2, 4, and 6 in Table 1.) Sharing their experiences about rotations would resemble the story sharing used elsewhere to promote healthcare student collaboration [48,49].

## 5. Limitations

A total of only 144 Medical Humanities students were studied at a single university. Thus, our data may seem difficult to generalize to similar programs at other universities. Nevertheless, our findings [7,8] were replicated with four separate cohorts of prospective medical students, and even during a pandemic and using remote learning. This reproducibility supports the notion that similar results would emerge if our methods were applied to other programs at Rocky Vista University.

Likewise, team community service promoted critical reflection and greater compassion in about 500 healthcare professional students enrolled elsewhere in Biochemistry courses [9,10]. These results bolster the proposition that our methods can be successfully applied at other universities. We encourage readers to further test the possibility that teams of prospective healthcare providers (1) use shared provocative experiences to build trust and psychological safety with one another and (2) exhibit frequent critical reflection to mitigate implicit biases and become more compassionate when given opportunities to make meaning of the experiences for themselves.

## 6. Conclusions

Most students experienced mental difficulties from selecting and performing team service-learning projects regardless of whether learning was remote or in person. This dissonance also arose from team and class discussions about the difficulties of communication, implicit association test results, and a pandemic. These mental difficulties generated critical reflection, mitigation of biases, and compassion in almost every student. Students’ reflective capacity and cognitive empathy scores also increased during our course. These discussions helped most students to realize that “unconscious biases might influence some of (their) clinical decisions and behaviors as healthcare professionals.” Even though learning was remote, students benefited from the trust, support, and psychological safety that developed through their work together in courses in their program. Finally, when learning was in person, students studied for all their courses with more interest owing to the relationships they saw between community service and the informational content of the courses (item 8 of Table 1 for 2019).

## Figures and Tables

**Table 1:** Team- and service-learning survey responses by Medical Humanities students in 2020 versus the previous three years (2017–2019). Except for item 8 in 2017, 2018, and 2020, median responses (not including outliers) were not 4.0. (That is, they were not neutral, one-sample Wilcoxon tests, *p* = 0.000.) Also shown are *p* values for the differences between responses in 2020 versus 2017–2019 (unpaired Mann–Whitney tests) and adjacent years for item 8 (Kruskal–Wallis test with multiple comparisons). * denotes statistically significant outliers.

1	2	3	4	5	6	7
Strongly Disagree	Disagree	Somewhat Disagree	Neither Agree/Disagree	Somewhat Agree	Agree	Strongly Agree
						*Median (no outliers)*
1	Having a team service-learning project in Medical Humanities was very engaging.					
***2017–2019***						***7***
1 (1.2%) *	1 (1.2%) *	1 (1.2%) *	2 (2.4%) *	12 (14.5%)	25 (30.1%)	41 (49.4%)
***2020***					*p* = 0.09	***6***
3 (5.1%) *	5 (8.5%) *	2 (3.4%)	0	14 (23.7%)	13 (22.0%)	22 (37.3%)
	In 2020, 22 students commented that they were disappointed because they could not serve in person, while 30 students indicated that their team service-learning was fulfilling/important.					
2	I would have been better off on another team in Medical Humanities.					
***2017–2019***						***1***
48 (57.8%)	22 (26.5%)	7 (8.4%)	4 (4.8%) *	2 (2.4%) *	0	0
***2020***					*p* = 0.000	***1***
48 (82.8%)	3 (5.2%) *	2 (3.4%) *	3 (5.2%) *	1 (1.7%) *	1 (1.7%) *	0
	In 2020, five students commented that it was difficult to engage with their teammates via Zoom or similar means, while 40 students indicated that they loved their team.					
3	Next year, Medical Humanities should continue to expect teams of MSBS students to perform service-learning projects and to write reflections on their experiences with the projects.					
***2017–2019***						***7***
1 (1.2%) *	0	1 (1.2%) *	1 (1.2%) *	4 (4.8%)	30 (36.1%)	46 (55.4%)
***2020***					*p* < 0.05	***7***
1 (1.7%)	1 (1.7%)	3 (5.0%)	5 (8.3%)	11 (18.3%)	7 (11.7%)	32 (53.3%)
	In 2020, 14 students questioned whether service-learning should be expected if it must be performed remotely, while 29 students commented that service-learning was valuable/helpful.					
4	All things considered, I could not have been assigned to a stronger team in Medical Humanities.					
***2017–2019***						***7***
0	2 (2.4%) *	2 (2.4%) *	12 (14.5%) *	7 (8.4%)	19 (22.9%)	41 (49.4%)
***2020***					*p* = 0.97	***7***
0	1 (1.7%) *	2 (3.4%) *	3 (5.1%)	8 (13.6%)	8 (13.6%)	37 (62.7%)
5	I gained very little from our service-learning project and written reflections on the project.					
***2017–2019***						***1***
43 (51.8%)	30 (36.1%)	7 (8.4%)	2 (2.4%) *	0	0	1 (1.2%) *
***2020***					*p* = 0.13	***1***
37 (63.8%)	10 (17.2%)	5 (8.6%)	1 (1.7)	5 (8.6) *	0	0
6	Medical Humanities should continue to use team-based learning in future courses.					
***2017–2019***						***7***
0	0	0	3 (3.6%) *	3 (3.6%) *	20 (24.1%) *	57 (68.7%)
***2020***					*p* > 0.99	***7***
0	0	0	2 (3.3%) *	6 (9.8%) *	9 (14.8%) *	44 (72.1%)
7	Writing reflections on our service-learning project fostered my professional development.					
***2017–2019***						***6***
1 (1.2%) *	0	2 (2.4%)	5 (6.0%)	17 (20.5%)	32 (38.6%)	26 (31.3%)
***2020***					*p* = 0.34	***6***
0	3 (4.9%)	1 (1.6%)	7 (11.5%)	16 (26.2%)	12 (19.7%)	22 (36.1%)
8	Encounters with people in our service-learning project caused me to study for all of my courses with more interest than likely would have occurred without the project.					
***2017***						***4***
3 (11.5%)	0	4 (15.4%)	9 (34.6%)	4 (15.4%)	4 (15.4%)	2 (7.7%)
***2018***					*p* = 0.83	***4***
1 (3.8%)	2 (7.7%)	1 (3.8%)	12 (46.2%)	6 (23.1%)	3 (11.5%)	1 (3.8%)
***2019***					*p* < 0.05	***6***
0	2 (6.7%)	2 (6.7%)	4 (13.3%)	6 (20.0%)	7 (23.3%)	9 (30.0%)
***2020***					*p* < 0.05	***5***
5 (9.6%)	4 (7.7%)	2 (3.8%)	11 (21.2%)	15 (28.8%)	5 (9.6%)	10 (19.2%)
	In 2020, 12 students indicated that remote/difficult/absent service during COVID-19 prevented this effect, while 11 students stated that this effort helped them stay interested, and three said it helped their team development.					
9	Encounters with people in our service-learning project will help me to be engaged with people regardless of the setting or disposition of the person.					
***2017–2019***						***6***
2 (2.4%) *	0	0	4 (4.8%)	12 (14.5%)	32 (38.6%)	33 (39.8%)
***2020***					*p* < 0.01	***6***
1 (1.8%)	2 (3.5%)	0	8 (14.0%)	14 (24.6%)	17 (29.8%)	15 (26.3%)
	In 2020, six students indicated that in-person engagement was not possible owing to the COVID-19 epidemic, while 18 students commented that service-learning helped them engage/communicate.					
10	Encounters with people/venues in our service-learning project helped me to see my potential biases toward people/venues more clearly.					
***2017–2019***						***6***
1 (1.2%) *	0	0	2 (2.4%) *	16 (19.3%)	26 (31.3%)	38 (45.8%)
***2020***					*p* < 0.01	***6***
0	4 (7.4%)	3 (5.6%)	5 (9.3%)	13 (24.1%)	11 (20.4%)	18 (33.3)
	In 2020, seven students commented that in-person encounters were not possible owing to the COVID-19 epidemic, while 19 students indicated that their reflections on service-learning, along with taking implicit association tests, helped them see their biases.					
11	Unconscious bias might affect some of my clinical decisions or behaviors as a healthcare professional.					
***2019 only***						***6***
0	1 (3.2%) *	0	1 (3.2%) *	2 (6.5%)	15 (48.4%)	12 (38.7%)
***2020***					*p* < 0.01	***5***
2 (3.3%)	1 (1.7%)	3 (5.0%)	5 (8.3%%)	21 (35.0%)	8 (13.3%)	20 (33.3%)
	In 2020, one student indicated that unconscious bias will not affect their behaviors as a healthcare professional, while 30 students said implicit bias will affect their behaviors, but they will work to control the bias and behaviors.					

**Table 2:** Categories of student biases revealed by the question “Of what biases did you become aware during encounters with people/venues in your service-learning project?” (56 of 61 students expressed at least one bias).

Negative Bias Category	Number of Students Revealing the Bias
Ageism (older or younger)	10
Economic class/homelessness	8
Obesity	7
Sexual orientation	7
Race	3
Substance abuse/addiction	3
Strong political opinions	3
Men	3
None/no interaction	3
Disabled	2
Mental health issues	2
Veterans	2
Favor same as me	2
Appearance/dress	1
Smokers	1
Women	1
Favor CNAs	1
End of life care	1

## Data Availability

All data are contained within the article.
